# Agency and shared decision-making in psychosis: A discourse analytic study

**DOI:** 10.1192/j.eurpsy.2026.10916

**Published:** 2026-07-31

**Authors:** K. N. S. Schriver, N. Buus

**Affiliations:** 1 Nordic languages and literature; 2 Public Health, Aarhus University, Aarhus, Denmark

## Abstract

**Introduction:**

Clinicians have often been criticised for excluding psychotic patients from decisions about their own treatment. While shared decision-making (SDM) is widely promoted, its use in psychiatry is still under-researched and sometimes contested. Some studies suggest SDM hasn’t delivered the expected benefits in general healthcare and may even leave patients feeling abandoned. This makes it important to look more closely at which kinds of SDM actually help patients feel more in control and empowered—especially in an inpatient, psychosis setting, where agency can be harder to support.

**Objectives:**

This study explores how clinicians can foster agency in psychotic patients during treatment planning. We focus on inpatient consultations where patients actively express a desire to be involved in treatment decisions.

**Methods:**

The study draws on four months of ethnographic fieldwork by the first author (KS) in a Danish mental health ward. During this time, she observed and/or recorded 58 doctor-patient conversations. For this analysis, we zoom in on consultations where patients showed a wish to participate in treatment decisions, using discourse analysis to unpack how these moments unfolded.

**Results:**

In the inpatient setting, patients mainly tried to be involved in decisions about hospitalisation status—sometimes indirectly—while clinicians tended to focus on decisions about medication. Clinician responses to patient agency fell into three broad patterns:

1. Ignoring the patient’s expressed or insinuated wish for treatment.
2. Acknowledging the wish but discouraging agency by asking the patient to put trust in the clinicians or medicine.
3. Fostering agency by identifying patient resources and encouraging patients to reflect on them.

**Conclusions:**

Clinicians can better support patient agency by 1) recognising patients’ treatment preferences and 2) actively identifying and discussing their coping strategies and goals. As psychiatric hospitals face challenges with frequent readmissions, helping patients mobilise their own resources may be one way to improve long-term outcomes.

**Disclosure of Interest:**

None Declared

